# Quality of life in children with cochlear implants in Kazakhstan

**DOI:** 10.1186/s12887-022-03254-w

**Published:** 2022-04-11

**Authors:** Ruslan Zhumabayev, Galiya Zhumabayeva, Gulnara Kapanova, Nailya Tulepbekova, Anuar Akhmetzhan, Andrej Grjibovski

**Affiliations:** 1 Faculty of Medicine and Health Care, Al-Farabi Kazakh National University, Almaty, Kazakhstan; 2 Kazakhstan Scientific Center of Anti-Infectious Drugs, Nur-Sultan, Kazakhstan; 3 Department of audiology, City Clinical Hospital No. 5, Almaty, Kazakhstan; 4 Northern State Medical University, Arkhangelsk, Russia; 5 I.M. Sechenov First Moscow State Medical University (Sechenov University), Moscow, Russia; 6 West Kazakhstan Marat Ospanov Medical University, Aktobe, Kazakhstan

**Keywords:** Kazakhstan, Cochlear implantation, Children, Quality of life

## Abstract

**Background:**

Although cochlear implantation (CI) has been performed in Kazakhstan since 2007 little is known about quality of life of patients after CI**.** The aim of this study was to assess the health-related quality of life (HRQoL) of Kazakhstani children after CI.

**Methods:**

Altogether, 53 families with a child using a cochlear implant for at least 1 year participated in the study between July 20, 2019 and February 20, 2020 at the Audiological Сenter of Almaty, Kazakhstan. The parents/caregivers completed the “Children with Cochlear Implants: Parental Perspectives (CCIPP)” questionnaire.

**Results:**

‘Well-being and happiness’ subdomain of the HRQoL yielded the highest ratings. ‘Communication’, ‘general functioning’, ‘self-reliance’, and ‘supporting the child’ subdomains each achieved significant (*p* < 0.01) associations with all HRQoL subdomains. There were positive correlations between language used by the parent who completed the questionnaire (Kazakh or Russian) and three HRQoL subdomains, including ‘well-being and happiness’, ‘supporting the child’ and ‘social relations’.

**Conclusion:**

Parents/caregivers reported high quality of life in all HRQoL subdomains. Further research in this area with more detailed socio-demographic and medical history data is required to identify quality of life predictors in children after cochlear implantation.

## Introduction

Cochlear implantation (CI) is widely used to treat children and adults with deafness or severe-to-profound hearing loss [1–3]. The increasing number of CI operations as a treatment option including children are due to quality of life improvement and a widespread roll out of newborn screening [3–7]. Early detection of deafness or severe-to-profound hearing loss with follow-up CI leads to better results with subsequent rehabilitation [3, 4].

Rehabilitation of children with implants includes medical, social, psychological and pedagogical components. For more than three decades of cochlear implantation use, at least ten questionnaires were developed, validated, and implemented to evaluate health-related quality of life (HRQoL) [5–13]. These varied by the number of areas evaluated, such as communication, self-reliance, social relations, education, speech perception, understanding of words, and pronunciation of words [8, 11, 14]. Open, closed, and mixed type of questionnaires are also used, which complicates or, on the contrary, simplifies information collection. Questionnaires have been developed for children of different age, parents/caregivers, even for specialists in this field, which allows to assess the development of children with implants comprehensively [14–18]. The choice of an instrument for a study of the HRQoL of children with CIs depends on the study aim.

“Children with Cochlear Implants: Parental Perspectives” (CCIPP) questionnaire does not require an interviewer and can be used anonymously, but does take time to fill it out. Fortunato-Tavares et al. translated, culturally adapted, and used CCIPP as the main tool to assess HRQoL of children with implants in Brazil [19] as did Byčkova et al. in Lithuania [20], Huttunen et al. in Finland [21], and Zhao et al. in China [22]. Kumar et al. published a study in children with implants in the United States [23]. These studies elucidated the influence of socio-demographic indicators, such as age of the child at assessment, age at implantation, duration of CI experience, parental educational level, language, and attendance at special educational institutions or schools for the deaf [14, 19–23].

CI as a method to treat children and adults with deafness or severe-to-profound hearing loss became available in the post-Soviet republics of Central Asia in early 2000’s [24] with the first CI in Kazakhstan in 2007 [24, 25], in 2009 in Uzbekistan, in the early 2010’s in Tajikistan, in 2014 in Kyrgyzstan, and in 2020 in Turkmenistan. HRQoL of children after CI, however, is almost never reported.

During its 13-year use in Kazakhstan [25], approximately 2000 CIs were performed, including 1200 in children. These operations are carried out in three of the country’s specialized medical centers with at least 100 taking place each a year [25]. According to the latest Kazakhstan law, newborns who have been diagnosed with deafness are eligible for CI only unilaterally free of charge, but bilaterally a year later. In general, current criteria for cochlear implantation in Kazakhstani children are:

1. severe-to-profound bilateral sensorineural hearing loss;
2. bilateral deafness;
3. auditory neuropathy with the ineffectiveness or low efficiency of hearing aids;
4. low efficiency of hearing aids.

Previously, in Kazakhstan there were no strict selection criteria for CI regarding the age for children with congenital and acquired deafness. This was probably due to the late implementation of newborn screening and the lack of parents’ alertness. All this led to late detection of hearing impairment and failure to determine the origin of deafness, and, consequently, to late CI. The current policy in Kazakhstan is to proceed with CIs in children under two years of age where possible. In Kazakhstan, only three centers offer social, auditory, psychological, and pedagogical rehabilitation and are located in the two largest cities of Kazakhstan, limiting the access to CI for families living in more remote areas. Rehabilitation includes the involvement of an audiologist, teacher of the deaf, a psychologist, an ENT doctor and a speech therapist. For children with deafness or hearing loss, as well as after CI, there is a special commission that assigns them to a specialized kindergarten or specialized school, according to age. Children after CI first go to a specialized kindergarten or school, and upon reaching a certain level of hearing and speech development, transfer to a regular school, that has an inclusive education class for children after CI or with hearing aids. In addition, these children can participate in the summer camps for two weeks every year. The inclusive education programs are being developed and include teaching children in non-specialized general education classes within mainstream schools if the child demonstrates sufficient hearing, language and speech development post-CI or with hearing aids. Eligibility for inclusive classes is determined by a special commission.

Kazakhstan is a bi-lingual country, Kazakh and Russian languages are used almost equally. Following the collapse of the Soviet Union, the young tend to report Kazakh as their first language, whereas older generations are more fluent in Russian. Therefore most studies in Kazakhstan are conducted in both Kazakh and Russian [26, 27].

As CI outcomes in children in Kazakhstan have never previously been reported, the first aim of the present study was to report HRQoL for children with implants. Specifically, it was hypothesized that HRQoL ratings would be positive and would exceed ratings of three out of five on the 5-point Likert scale. The second aim was to examine associations between CI-specific HRQoL subdomains. The third aim was to identify the associations between child/family socio-demographic variables and parent/caregiver ratings of CI-specific HRQoL subdomains for children with implants. Specifically, it was hypothesized that the child age at CI activation would be associated with higher ratings on the HRQoL subdomains and that the use of Kazakh and Russian language used by the parent who completed the questionnaire would be associated with HRQoL subdomains.

## Materials and methods

### Sample and procedure

**Fig. 1:**
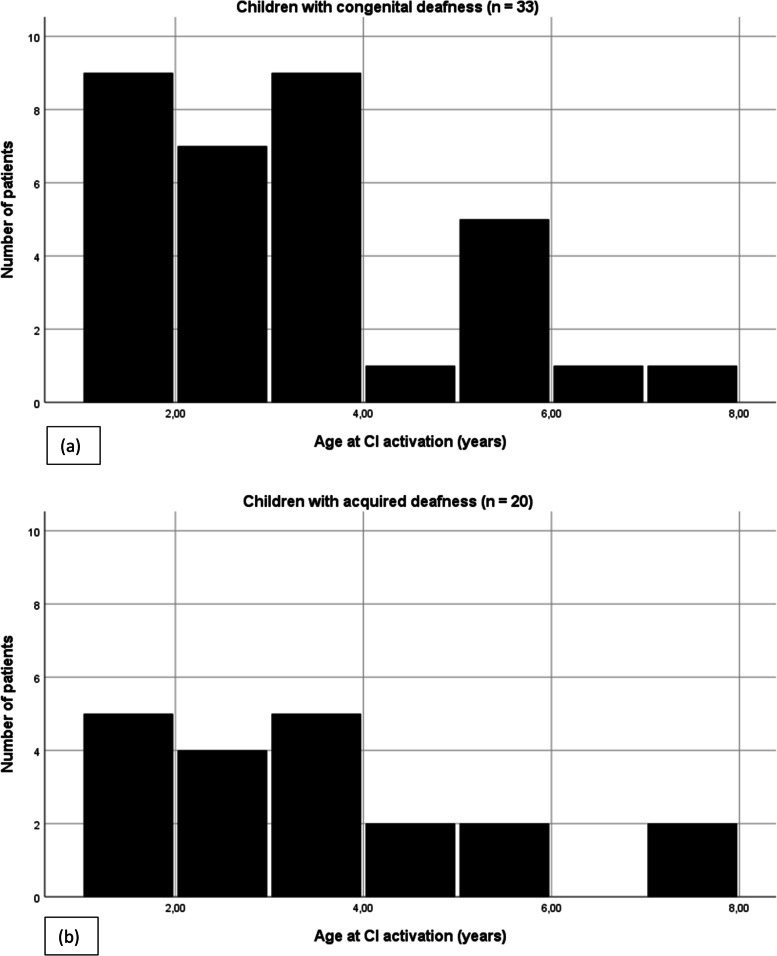
Age at CI activation with regard to the origin of deafness. **a** Represents the age at CI activation of children with congenital deafness. **b** Represents the age at CI activation of children with acquired deafness

The sample involved parents/caregivers of children (child age at study mean = 7.33, SD = 3.28, range = 2.8-15) who attended CI follow-up care and consulted an audiologist at a time of this study. Altogether, 53 families with their children (73.6% males, 62.3% congenital hearing loss, 92.5% unilateral CI) who had used cochlear implants for at least one year participated in this study. The study was conducted between July 20th 2019 and February 20th 2020 in the Audiological Сenter of Almaty, Republic of Kazakhstan. Parents/caregivers independently (without any help of the audiologist or an interviewer) completed the questionnaire. Written informed consent was obtained from all parents/caregivers who agreed to participate in this study. Furthermore, parents/caregivers were asked some additional information related to their family and child, see Tables 1, and 2 and Fig. 1. The variables were categorized in the following way: age at assessment, age at CI activation and duration of CI was treated as continuous variables. Gender was coded; 1 for ‘male’ and 2 for ‘female’. Parental educational level was categorized into two groups; ‘high school’ for 2 and ‘university’ for 1. Attendance at school for the deaf was coded; 0 for ‘no’ and 1 for ‘yes’. Attendance at special educational institutions was coded; 0 for ‘no’ and 1 for ‘yes’. CI surgery was coded; 1 for ‘unilateral’ and 2 for ‘bilateral’. Participants, who completed the questionnaire were coded; 1 for ‘father’, 2 for ‘mother’, 3 for ‘grandmother’ and 4 for ‘caregiver’. Etiology of deafness was categorized; 1 for ‘congenital’ and 2 for ‘acquired’. Language (language used by the parent who completed the questionnaire) coded; 1 for ‘Kazakh’ and 2 for ‘Russian’.

**Table 1:** Socio-demographic characteristics of the study participants (*N* = 53)

	% (n)
Gender	
Male	73.6 (39)
Female	26.4 (14)
Cochlear Implantation	
Unilateral	92.5 (49)
Bilateral	7.5 (4)
^a^Etiology of deafness	
Congenital	62.3 (33)
Acquired:	
Ototoxic antibiotics	20.8 (11)
Acute respiratory viral infections	3.7 (2)
Unknown reasons	13.2 (7)
^a^School for the Deaf before cochlear implantation	11.3 (6)
Not attended to the school for the Deaf	88.7 (47)
^a^ Special educational institutions at the moment of assessment:	
Kindergarten	20.8 (11)
Boarding school	43.4 (23)
Speech therapy center	7.5 (4)
Not attended any educational institution or school at the moment of assessment	28.3 (15)
^a^Parental education level	
High school	49.1 (26)
University	50.9 (27)
Participant, who completed the questionnaire	
Mother	83 (44)
Father	11.3 (6)
Grandmother	3.8 (2)
Caregiver	1.9 (1)
Spoken language (language of the questionnaire)	
Kazakh	47.2 (25)
Russian	52.8 (28)

^a^Parent-reported data

**Table 2:** Socio-demographic information of children with cochlear implants

	Total (*N* = 53)			Congenital deafness (*n* = 33)			Acquired deafness (*n* = 20)		
	Mean	SD	Range	Mean	SD	Range	Mean	SD	Range
Age at CI activation (years)	3.17	1.62	1-7	3.08	1.55	1.08-7	3.3	1.77	1-7
Age at assessment (years)	7.33	3.28	2.8-15	7.78	3.28	2.8-15	6.58	3.23	3-15
Duration of CI experience (years)	4.21	2.45	1-12	4.79	2.61	1-12	3.26	1.86	1.2-8

*CI* Cochlear implant

Taking into account that some families might use both Kazakh and Russian languages equally in daily life, the language in which the questionnaire was filled out was considered to be the language of the family. All methods were carried out in accordance with relevant guidelines and regulations.

This research involved participants from the all regions of Republic of Kazakhstan. The exclusion criteria were: children using an implant for less than one year, and non-citizens of Republic of Kazakhstan. In Kazakhstan, children are considered as persons under the age of 18. Al-Farabi Kazakh National University Review Board approved this study (IRB – A060). Participants of this research received no compensation for their input.

### Assessment Tool

The HRQoL questionnaire, Children with Cochlear Implants: Parental Perspectives was filled out by the parents/caregivers and had been widely used in the previous studies [14, 19–23, 28, 29]. The Children with Cochlear Implants: Parental Perspectives is a validated, approved, CI-specific, closed-set questionnaire based on parental responses. It includes 74 statements covering two domains of the process of CI: decision-making (26 items) and outcomes of implantation (48 items). The outcomes of implantation domain consist of eight subdomains, including six child-related subdomains (‘communication’, ‘general functioning’, ‘well-being and happiness’, ‘self-reliance’, ‘social relations’, ‘education’) and two family-related subdomains (‘effects of implantation’ and ‘supporting the child’) (see Table 3 for subdomain descriptions and sample items). The CCIPP questionnaire is intended for children using the cochlear implant more than one year. This questionnaire was translated and adapted into Kazakh and Russian. The translation procedure consisted of direct translation by two independent translation agencies and a reverse translation by another two independent translation agencies. An audiologist then generated the final version of translated questionnaire. Direct translation from Kazakh to English and reverse translation were performed without intermediary translation to Russian.

**Table 3:** Cochlear implant–specific quality of life subdomains

Subdomain (Items)	Description	Sample Item
Communication (6)	Ease, quality, and quantity of communication and conversation	Communication is difficult even with people he knows well.
General functioning (6)	Changes in attention, safety, and engagement	I can now let her play outside as she is aware of the sound of traffic.
Well-being (5)	Happiness and frustration	He continues to be a happy child and good fun to be with
Self-reliance (4)	Indicators of confidence and independence	A significant change has been improvement in her confidence
Social relations (7)	Relationships within and outside the family	He does not make friends easily outside the family.
Education (7)	Performance of the child at school; placement and responsiveness within the school district	The local school and support services adequately meet all our needs concerning the use of her implant at school
Effects of implantation (7)	Progress with the cochlear implant, future concerns regarding device function, and child reaction to the device	I worry that he will blame me for my decision for him to have an implant.
Supporting the child (6)	Amount and effects of help required by child before and after implantation	A parent of a child with an implant needs to be patient as benefits may take time to show.

This table includes cochlear implant–specific quality of life subdomains in the Children with Cochlear Implants: Parental Perspectives questionnaire. Description according to Archbold et al. [28]; Kumar et al. [23]

### Statistical analysis

Parents/caregivers rated their responses to each statement on a Likert 5-point scale: strongly agree (coded as 5), agree (4), neither agree nor disagree (3), disagree (2), and strongly disagree (1). Twenty-eight statements were phrased in a negative and 46 in a positive way. Negative statements scoring was reversed to achieve meaningful statistical representation, whereas the higher ratings represented better HRQoL. At the stage of filling out the questionnaires we made sure there were no missing values. We calculated means and standard deviations for all variables of interest. Spearman correlation coefficients (rho) were calculated to assess the associations between HRQoL subdomains between socio-demographic variables, including age at assessment of the child, age at implantation, and duration of CI use, and the mean HRQoL subdomains ratings. Mann-Whitney *U* test was computed to examine the information between family and child (gender, parental educational level, attendance at special educational institutions or school for the deaf, language of questionnaire, etc.) and the mean HRQoL subdomains. Statistical analyses were performed using SPSS software, v. 23 (IBM Corporation; Chicago, IL, USA).

## Results

### Comparisons between health-related quality of life subdomains

**Fig. 2:**
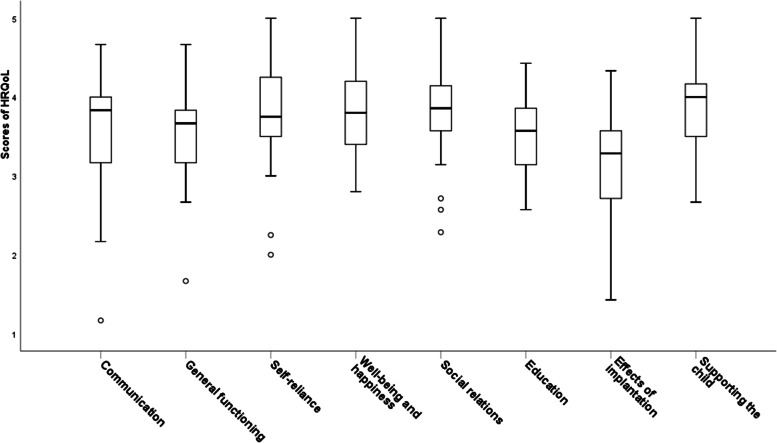
Parental ratings of HRQoL subdomains of their child with CI

Mean ratings were greater than three for all eight HRQoL subdomains on a 5-point Likert scale (mean = 3.65, SD = 0.4, range = 2.31–4.52), indicating that parents considered their child’s HRQoL as either average or being more positive than negative. ‘Well-being and happiness’ received the highest ratings (mean = 3.89, SD = 0.5, range = 2.8–5.0), followed by ‘supporting of the child’ (mean = 3.87, SD = 0.48, range = 2.67–5), ‘social relations’ (mean = 3.85, SD = 0.52, range = 2.29–5.0) and ‘self-reliance’ (mean = 3.80, SD = 0.64, range = 2.0–5.0) as shown in Fig. 2. ‘Communication’ (mean = 3.58, SD = 0.73, range = 1.17–4.67), ‘general functioning’ (mean = 3.57, SD = 0.51, range = 1.67–4.67) and ‘education’ (mean = 3.49, SD = 0.45, range = 2.57–4.43) also rated positive. The ‘effects of implantation’ received the least positive ratings (mean = 3.15, SD = 0.6, range = 1.43–4.33).

### Associations between health-related quality of life subdomains

**‘**Communication’, ‘general functioning’, ‘self-reliance’, and ‘supporting the child’ were each significantly associated with all HRQoL subdomains.

Table 4 presents all correlations between HRQoL subdomains.

**Table 4:** Spearman’s correlation coefficients for the associations between health-related quality of life subdomains in Kazakhstani children (*N* = 53)

Subdomain	Communication	General functioning	Self-reliance	Well-being and happiness	Social relations	Education	Effects of CI	Supporting the child
Communication	-	0.483*	0.615*	0.468*	0.513*	0.457*	0.607*	0.362*
General functioning			0.534*	0.385*	0.488*	0.424*	0.384*	0.451*
Self-reliance				0.442*	0.557*	0.364*	0.417*	0.370*
Well-being and happiness					0.332	0.266	0.391*	0.405*
Social relations						0.315	0.327	0.351*
Education							0.445*	0.317*
Effects of CI								0.422*
Supporting the child								-

*p < 0.01

*CI* Cochlear implant

### Associations between socio-demographic variables and health-related quality of life ratings

Analysis of the sample (*N*=53) indicated age at assessment was not significantly correlated with any of the eight HRQoL subdomains. Age at CI activation and duration of CI use were also not significantly correlated with any of the eight HRQoL subdomains. In order to examine onset of hearing loss, the groups were divided into those with congenital deafness (*n*=33) and those children with acquired (*n*=20) deafness. For the group of children with acquired deafness, only age at assessment was significantly positively correlated with ‘general functioning’, ‘effects of implantation’ and ‘supporting the child’ (*p* < 0.01). Table 5 presents all correlations between above-mentioned variables and HRQoL subdomains. Duration of CI use was significantly correlated with age at assessment, so was not included in Table 5 and excluded from further analysis.

**Table 5:** Spearman’s correlation coefficients for the associations between socio-demographic factors and parental assessment of children’s quality of life in Kazakhstan

Subdomains	Total (*N* = 53)		Congenital deafness (*n* = 33)		Acquired deafness (*n* = 20)	
	Age at assessment	Age at CI activation	Age at assessment	Age at CI activation	Age at assessment	Age at CI activation
Communication	0.078	-0.058	0.023	0.052	0.263	-0.140
General functioning	0.309	0.129	0.111	0.098	0.588^*^	0.209
Self-reliance	0.167	0.076	-0.118	0.021	0.486	0.206
Well-being and happiness	0.030	-0.183	-0.041	-0.205	0.087	-0.194
Social relations	0.152	-0.005	0.028	0.137	0.321	-0.159
Education	0.297	0.090	0.351	0.213	0.069	-0.032
Effects of implantation	0.055	-0.060	-0.140	-0.157	0.607^*^	0.099
Supporting the child	0.186	0.161	0.053	0.136	0.595^*^	0.213

*p < 0.01

*CI* Cochlear implant

*p*-values in parenthesis

We identified correlations between language used by the parent who completed the questionnaire and three HRQoL subdomains including ‘well-being and happiness’ (Mann-Whitney test, *p* = 0.004), ‘supporting the child’ (Mann-Whitney *U* test, *p* = 0.008), and ‘social relations’ (Mann-Whitney *U* test, *p* = 0.039). Parents/caregivers of children with CIs who spoke Kazakh (mean = 3.99, SD = 0.54) showed a higher rating in ‘social relations’ than those who spoke Russian (mean = 3.72, SD = 0.47). Parents/caregivers of children with CIs who spoke Russian (mean = 4.08, SD = 0.55) had higher ratings of ‘well-being and happiness’, than those who spoke Kazakh (mean = 3.69, SD = 0.42). Parents/caregivers of children with CIs who spoke Russian (mean = 4.04, SD = 0.37) showed a higher rating in ‘supporting the child’, than those who spoke Kazakh (mean = 3.68, SD = 0.53). There were no statistically significant correlations between other socio-demographic variables, such as gender, parental education level and attendance at special educational institutions at the time of assessment and any of the eight HRQoL subdomains.

## Discussion

Parents/caregivers positively rated all eight subdomains (ratings exceeded three points out of five). The ‘well-being and happiness’ subdomain received the highest rating and the ‘effect of implantation’ subdomain received the lowest rating. ‘Communication’, ‘general functioning’, ‘self-reliance’, and ‘supporting the child’ were significantly correlated with all HRQoL subdomains. Therefore, a high rating in one subdomain implied a positive rating in another. With regard to socio-demographic variables there were statistically significant correlations between the language used by the parent who completed the questionnaire and the following HRQoL subdomains: ‘social relations’, ‘well-being and happiness’, and ‘supporting the child’.

### Comparison with other studies with CCIPP

In all studies, using the CCIPP questionnaire, the subdomains achieved more than three points and the result was rated as positive [7, 14, 19–23, 28, 29]. The present study hypothesis that HRQoL subdomains were generally positive was supported (mean ratings for all eight HRQoL = 3.65). The highest three subdomains were ‘well-being and happiness’, ‘supporting the child’, and ‘social relations’ which obtained ratings of 3.89, 3.87 and 3.85 respectively. In contrast, Kumar and colleagues (2015) obtained 3.65 for ‘well-being and happiness’; their highest rating was obtained for ‘communication’ with 3.93 [23]. Zhao et al. [22] and Byčkova et al. [20] reported the higher rating in ‘social relations’ with 3.72 and 4.05 respectively [20, 22].

Moreover, Kumar et al. (2015) noted that the high ratings in ‘communication’, ‘general functioning’ and ‘social relations’ subdomains were due to the filling out of questionnaires by parents/caregivers in the summer camp, where the children with implants were gathered together and professionals worked with them [23]. The same subdomains had a high level in the study of Huttunen et al. (2009), but children after CI with concomitant pathology were included in the sample [21] making direct comparison difficult. Zhao et al. [22] reported a low rating (3.45) in the ‘communication’ subdomain, consistent with the present study (3.59) [22]. Zhao et al. [22] speculated that CIs were used for a shorter period than in the study by Kumar et al. (2015) [22, 23]. In the present study the duration of CI use was 4.21 years, while in the study by Kumar et al. (2015) it was 7.47 years and 3.7 years in Byčkova et al. (2018) study [20, 23].

The lowest rating in the present study was in the ‘effects of implantation’ subdomain (3.15), which was consistent with: Kumar et al. (2015) (3.11); Byčkova et al. (2018) (3.16) [20] and Huttunen et al. (2009) (3.7) [20, 21, 23]. Low ratings of this subdomain suggested that parents’ observation of their child post-CI did not reach their pre-implant expectations for communication outcomes. Differences in the study samples and the age range at CI and age range at survey make it challenging to account for differences in findings. Of note, ‘communication’ and ‘social relations’ also obtained high ratings in past studies assessing HRQoL for children with CIs [6, 8, 17, 30]. Further direct comparisons of the present study HRQoL subdomains findings with Brewis et al. [29], Byčkova et al. [20], Zhao et al. [22] and Kumar et al. (2015) are in Table 6 [20, 22, 23, 29].

**Table 6:** Means (M) and standard deviations (SD) for the main findings in this study in comparison with recent studies from other countries

Language	Present study	Brewis et al. 2020 [29]	Byčkova et al. 2018 [20]	Zhao et al. 2019 [22]	Kumar et al. 2015 [23]
	Kazakh and Russian	English	Lithuanian	Mandarin	English
Sample size	53	54	28	123	33
Range of age (years)	2.8-15	6.6-18.3	3.5-18.7	1.7-7.3	4-18
Age at assessment (years)	7.33 (3.28) ^a^	12.2 (3.6)	6.1 (3.3)	3.37 (1.20)	9.85 (3.30)
Age at CI activation (years)	3.17 (1.62)	3.90 (2.41)^b^	2.41 (2.25)	2.06 (1.08)	2.47 (1.85)
Duration of CI experience (years)	4.21 (2.45)	8.21 (4.10)	3.7 (1.3)	1.36 (0.53)	7.47 (2.80)
Communication	3.59 (0.73)	4.15 (0.62)	3.90 (0.77)	3.45 (0.71)	3.93 (0.62)
General functioning	3.57 (0.51)	4.05 (0.51)	^c^	3.62 (0.50)	3.86 (0.47)
Self-reliance	3.80 (0.64)	3.88 (0.63)	3.30 (0.27)	3.55 (0.47)	3.71 (0.77)
Well-being and happiness	3.89 (0.53)	3.81 (0.60)	^c^	3.70 (0.45)	3.65 (0.62)
Social relations	3.85 (0.52)	3.87 (0.52)	4.05 (0.41)	3.72 (0.43)	3.85 (0.38)
Education	3.49 (0.45)	3.70 (0.64)	^c^	3.38 (0.47)	3.32 (0.50)
Effects of implantation	3.15 (0.60)	3.49 (0.62)	3.16 (0.46)	3.67 (0.69)	3.11 (0.70)
Supporting the child	3.87 (0.48)	3.46 (0.47)	3.89 (0.49)	3.66 (0.59)	3.74 (0.56)

^a^Standard deviation (SD) in parenthesis

^b^(*n* = 40) only for age at CI activation, (*N* = 54) for others of Brewis et al. [29] study

^c^no information found in Byčkova et al. [20] study

*CI* Cochlear implant

Zhao et al. performed such table in 2019 for the first time. Full information for the reference: Brewis et al. [29], Byčkova et al. [20], Zhao et al. [22], Kumar et al. 2015

### Associations between HRQoL subdomains and socio-demographic variables

The associations between socio-demographic variables such as age at the time of the study, age at CI activation, duration of CI experience and psychosocial subdomains were not statistically significant in total sample (*N*=53). With regard to children with congenital deafness (*n*=33) there were no significant correlations between age at assessment, age at CI activation, duration of CI experience and any of eight HRQoL subdomains. Only age at assessment showed positive significant correlations with three psychosocial subdomains, including ‘general functioning’, ‘effects of implantation’, ‘supporting the child’ in children with acquired deafness (*n*=20). Dividing our sample into two smaller groups reduced statistical power and thus precludes making firm conclusions about HRQoL subdomains in our cohort. Due to the wide range in the age of child participants, presence/absence of child concomitant pathologies, differences in family socioeconomic status, greater/lesser parental participation in child development post-CI, and higher/lower parental education, past study findings regarding associations between child/family variables and child outcomes and quality of life vary [8, 10, 14, 21–23, 29, 30].

With respect to the family’s socio-demographic variables, the present study demonstrated a positive relationship between the HRQoL subdomains and the language used by the parent who completed the questionnaire. Mann-Whitney *U* test indicated a positive association between the language used by the parent who completed the questionnaire and ‘well-being and happiness’ (*p* = 0.004), ‘social relations’ (*p* = 0.039) and ‘supporting the child’ (*p* = 0.008). The ‘social relations’ subdomain received a high rating when the language used by the parent who completed the questionnaire was Kazakh. In contrast, ‘well-being and happiness’, and ‘supporting the child’ subdomains received a high rating when the language used by the parent who completed the questionnaire was Russian. This can be explained by the history and traditions of nations of the Central Asian region, who used to live in large families (including not only mother, father, children, but also grandfathers, grandmothers, cousins, uncles, aunts) [31]. This way of life is preserved to this day among the majority of the population of this territory. This mentality, intrinsic to the Kazakh people, includes the significance of relations between relatives and neighbors; also, at school, university and at work. In Kazakh-speaking families parents (and often other close relatives) instill in children the need to establish relationships with people around them [31]. Another aspect of this is that in Kazakh-speaking families there are often three or more children, so parents probably cannot devote more time to the child with implant (work, life, welfare of other children). The differences in family life for Kazakh-speaking versus Russian-speaking families may account for some of the differences in parent ratings of HRQoL subdomains observed in the present study. As past studies have not compared Kazakh-speaking versus Russian-speaking participants, this is a finding unique to the present study that requires replication in a larger cohort. Kumar et al. (2015) surveyed their participants in English and Brewis et al. [29] also had English as an inclusion criterion [23, 29]. Zhao et al. [22] measured the quality of life of children within a Mandarin language environment, whilst Huttunen et al. (2009) carried out their study in Finnish [21, 22]. As indicated above, Kazakhstan is a bilingual country where Kazakh and Russian languages are used equally, so understanding any potential differences in HRQoL subdomains is important.

In the present study, there were no statistically significant associations between socio-demographic variables such as gender, level of parents’ education, or attendance at specialized educational institutions by the child post-CI and the HRQoL subdomains. Huttunen et al. (2009) and Zhao et al. [22] also showed no statistically significant associations between the number of children in the family and HRQoL subdomains [21, 22].

### Strengths and limitations

This study is not without limitations. Firstly, the study was carried out in one center, and although participants were from all over the Republic of Kazakhstan, patients who visited the other two centers did not enroll in the study, thereby, the sample size was limited. Secondly, parents/caregivers filled out the questionnaire by themselves, which could affect the study. We could not verify our data with departmental records, because we could not gain access to them, which is another limitation of our study. A further limitation of the present study was the inability to distinguish between various causes of congenital anomalies, such as CMV, genetic, hypoxia, jaundice, given that congenital abnormalities is a very heterogenous group. Additional needs of children were not considered in this study, which is another limitation of our report. We also chose not to use other questionnaires, such as CAP, SIR, MAIS and MUSS which could also be acknowledged as a limitation. Additionally, the fact that private tutoring with children after CI, plays a large role in their development, it would be correct to integrate the socioeconomic status of parents/caregivers, which our study did not consider. It is also necessary to notice that the audiological screening of newborns in Kazakhstan started in 2010, hence, data on the etiology of deafness prior to 2010 may have been less accurate for 12 children (out of 53) who were ten years and older at the time of this study. The age range is too wide, which did not allow conclusions to be drawn by age groups and excluded the possibility of identifying significant confounding factors.

The primary strength of this study is that for the first time in Kazakhstan an assessment was conducted of the HRQoL of children after CI. The assessment instrument was a translated and culturally adapted questionnaire, CCIPP, which is widely and universally used to indicate the HRQoL of children after CI throughout the world.

## Conclusions

Likert ratings above three (out of five) suggested that parents/caregivers indicated that they were generally satisfied with all HRQoL subdomains; they rated ‘well-being and happiness’, ‘supporting the child’, and ‘social relations’ higher than ‘effects of implantation’ and ‘education’ subdomains.

With the exception of the independent variable ‘language used by the parent who completed the questionnaire’, there were no statistically significant associations between HRQoL subdomains and child variables (gender, age at assessment, age at CI activation, duration of CI experience) nor statistically significant associations between HRQoL subdomains and other socio-demographic variables (level of parents’ education, or attendance at specialized educational institutions by the child post-CI) in this small cohort. The variable ‘language used by the parent who completed the questionnaire’ was positively correlated with the ‘well-being and happiness’, ‘social relations’, and ‘supporting the child’ subdomains. These results suggested the importance of subtle differences in family culture and family life for children using CIs in the region under study. Further research in this field is required to identify patterns of family life in the Central Asia region that may be associated with CI outcomes and quality of life, but extrapolating from studies in Western countries, it is still possible to encourage families to provide abundant language opportunities for their child’s development [32]. Specialists, who work with CI recipients’ families, are recommended to consider child and family socio-demographic variables carefully in order to optimize the child’s use of their CIs.

## Data Availability

The datasets generated and analysed during the current study are available from the corresponding author on reasonable request.

## Abbreviations

CI: Cochlear Implantation
HRQoL: Health-related quality of life
CCIPP: Children with Cochlear Implants: Parental Perspectives questionnaire
